# Compression médullaire au cours d’une spondylodiscite tuberculeuse: à propos d’un cas

**DOI:** 10.11604/pamj.2018.31.101.17054

**Published:** 2018-10-10

**Authors:** Nabil Tiresse, Ahmed Abid

**Affiliations:** 1 Service Pneumologie, Hôpital Militaire d’Instruction Mohamed V, Rabat, Maroc

**Keywords:** Spondylodiscite, compression médullaire, Gene Xpert, décompression chirurgicale, Spondylodiscitis, spinal cord compression, Gene Xpert TB test, surgical decompression

## Abstract

We here report the case of a 76-year old patient admitted to Pulmonology Department with tuberculous spondylodiscitis diagnosed on the basis of clinical signs including weight loss, prolonged fever and stabbing lumbar pain occurred 2 months before. The diagnosis was confirmed by lumbar CT scan and by Genexpert TB test on lumbar fluid taken from the abnormal L1 vertebral body, which showed mycobacterium tuberculosis. Three weeks after the beginning of treatment the patient had motor deficit in lower limbs followed by sensory deficit. These abnormalities were confirmed by MRI which showed abnormal L1 somatic signal with collapse of the vertebral body (A). These lesions enhanced after gadolinium injection (B) that also showed posterior cortical rupture with epiduritis. Radiological examination was performed which confirmed the diagnosis of spinal cord compression associated with TB spondylodiscitis. A few days after, the patient had severe sepsis complicated by septic shock after which he died. Spinal compression is rare in patients with spondylodiscitis. It has a high potential for irreversible complications in the absence of early treatment for spinal cord decompression. Surgery and early antibacillar treatment ensure favorable outcome in the medium and long term.

## Image en médecine

Patient âgé de 76 ans, admis en pneumologie pour prise en charge d'une spondylodiscite tuberculeuse découverte par un tableau clinique associant amaigrissement, fièvre prolongée et douleur lombaire lancinante qui datent de 2 mois auparavant, confirmée par tomodensitométrie lombaire. Le diagnostic a été retenu suite à l'étude Gene Xpert sur liquide de ponction biopsie au niveau de l'anomalie vertébrale L1 qui a mis en évidence le mycobacterium tuberculosis. Trois semaines après début de traitement le patient a présenté un déficit moteur au niveau des 2 membres inférieurs suivi d'un déficit sensitif. Ces anomalies ont été confirmées sur IRM qui a montré des anomalies du signal somatique de L1 avec tassement du corps vertébral (A), ces lésions sont réhaussées après injection de gadolinium (B) qui a montré également une rupture corticale postérieure avec épidurite en regard. Cet aspect radiologique a permis de confirmer le diagnostic de compression médullaire sur spondylodiscite tuberculeuse. Quelques jours après le patient a présenté des signes de sepsis sévère compliqué d'un choc septique suite auquel le patient était décédé. La compression médullaire est l'un des aspects rares apparaissant au cours d'une spondylodiscite ayant un potentiel élevé de complications irréversibles en absence d'intervention précoce pour décompression médullaire. La chirurgie et le traitement antibacillaire démarré précocement sont garants d'une évolution favorable à moyen et à long terme.

**Figure 1:**
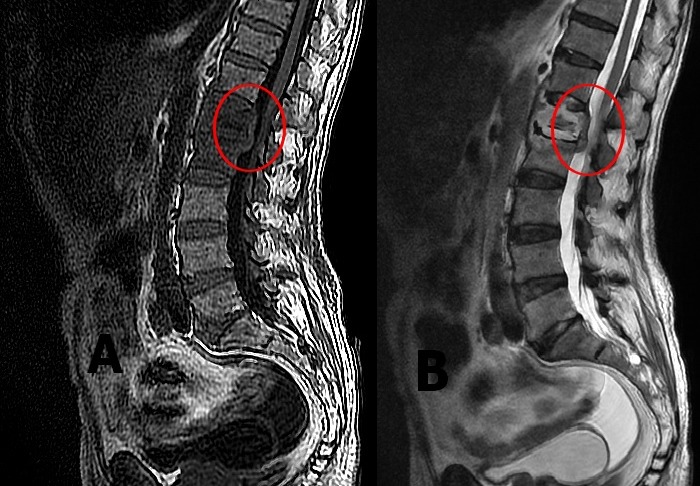
A): anomalie du signal somatique de L1 avec tassement du corps vertébral; B) réhaussement des lésions après injection de gadolunium

